# Membrane association and remodeling by intraflagellar transport protein IFT172

**DOI:** 10.1038/s41467-018-07037-9

**Published:** 2018-11-08

**Authors:** Qianmin Wang, Michael Taschner, Kristina A. Ganzinger, Charlotte Kelley, Alethia Villasenor, Michael Heymann, Petra Schwille, Esben Lorentzen, Naoko Mizuno

**Affiliations:** 1 Max Planck Institute of Biochemistry, Department of Structural Cell Biology, Am Klopferspitz 18, D-82152 Martinsried, Germany; 2 Max Planck Institute of Biochemistry, Department of Cellular and Molecular Biophysics, Am Klopferspitz 18, D-82152 Martinsried, Germany; 3 Department of Molecular Biology and Genetics, Aarhus University, Gustav Wieds Vej 10c, DK-8000 Aarhus C, Denmark; 4 Present Address: University of Lausanne, Department of Fundamental Microbiology, CH-1015 Lausanne, Switzerland; 5 Present Address: AMOLF, Living Matter Department, Science Park 104, 1098 XG Amsterdam, The Netherlands

## Abstract

The cilium is an organelle used for motility and cellular signaling. Intraflagellar transport (IFT) is a process to move ciliary building blocks and signaling components into the cilium. How IFT controls the movement of ciliary components is currently poorly understood. IFT172 is the largest IFT subunit essential for ciliogenesis. Due to its large size, the characterization of IFT172 has been challenging. Using giant unilamellar vesicles (GUVs), we show that IFT172 is a membrane-interacting protein with the ability to remodel large membranes into small vesicles. Purified IFT172 has an architecture of two globular domains with a long rod-like protrusion, resembling the domain organization of coatomer proteins such as COPI-II or clathrin. IFT172 adopts two different conformations that can be manipulated by lipids or detergents: 1) an extended elongated conformation and 2) a globular closed architecture. Interestingly, the association of IFT172 with membranes is mutually exclusive with IFT57, implicating multiple functions for IFT172 within IFT.

## Abstract (web summary)

Cilia formation requires Intraflagellar transport (IFT) to move ciliary building blocks and signaling components into the cilium. Here authors use in vitro reconstitution and electron microscopy on IFT172 and reveal its ability to remodel large membrane surfaces into small vesicles.

## Introduction

Eukaryotic cells are organized into different compartments and organelles. Cilia or flagella (interchangeable terms) protrude from the surface of most eukaryotic cells and serve important functions in motility, sensory reception and signaling^1^. Motile cilia are present in several copies on the surface of cells and beat in coordinated waves to create movement. It is less well known that most animal cells contain a primary cilium (in general non-motile) that is normally present in one copy per cell^1^. Primary cilia on mammalian cells were discovered at the end of the nineteenth century^2^, however, research in this area was largely neglected because of the view that eukaryotic primary cilia might only be vestigial and relatively unimportant organelles. This view has been changed by evidence that primary cilia function in sensory perception, important both during development and in normal physiology^2^. Cilia are important for a number of signaling pathways such as platelet-derived growth factor receptor α (PDGFRα), Sonic hedgehog (Shh), Epidermal Growth Factor (EGF), WNT and 5HT_6_ serotonin signaling^3^. Both sensory reception and signaling in the cilium is a result of clustering of receptors in the ciliary membrane. Besides the loss of sight and smell, defects in cilia can lead to many human genetic diseases collectively termed ciliopathies^4^. These include polycystic kidney disease, cognitive impairment, limb deformities, obesity, *situs inversus* (visceral organs have their position inverted), respiratory distress, ectopic pregnancy and sterility^4^. Understanding the molecular basis of the structure and function of cilia is thus crucial.

At the ultrastructural level, all cilia have a common architecture consisting of a microtubular axoneme surrounded by a membrane that is continuous with the cellular plasma membrane^1^. The axoneme is anchored in a barrel-shaped basal body and originates from a centriole^5^. The cilium consists of more than 600 different proteins including structural axonemal proteins such as tubulin subunits and dynein motors, but also small GTPases, receptors and ion channels, glycolytic enzymes and proteins directly involved in transport processes^6^. The proteome of primary cilia has been captured by cilia-targeted proximity labeling, elucidating ciliary export mechanisms of signaling components^7^. Moreover, flagellum elongation takes place by addition of new subunits at the distal end^8–10^. Consequently, proteins destined for the cilium are actively transported along the outer microtubules of the axoneme, a process termed intraflagellar transport (IFT)^11^. IFT is necessary for the assembly of cilia or flagella in almost all species studied so far, from protists to mammals. The process of IFT is mediated by a large ~22-subunit protein complex termed the IFT particle. Ciliary cargo is transported on large trains of IFT particles to the tip of the flagellum (anterograde transport) in a kinesin-II motor-dependent manner^12,13^ although the molecular basis for train formation is currently unknown. The IFT trains are sandwiched between the ciliary membrane and the axoneme suggesting that IFT proteins may have some affinity for membranes although no membrane-interacting domains are predicted in any IFT subunit^14^. IFT complexes are also shown to associate with various membrane systems of diverse functions. These include the well-studied association of IFT20 with the Golgi and with vesicles being transported from the Golgi to the cilium in mammalian cells^15^. Other studies demonstrated that IFT proteins associate with vesicles in ciliated and non-ciliated neurons^16^ as well as on vesicles at the cleavage furrow of dividing *Chlamydomonas* cells^17^. Additionally, IFT proteins were observed on vesicles destined for the cilium in *Chlamydomonas*^18^ and membrane shedding from cilia was observed for a number of organisms and cell types including *Chlamydomonas* and *C.elegans*^19–21^. These observations suggest that subunits of the IFT complex subunits may have affinity for membranes although the molecular mechanisms of such IFT-membrane interactions remain elusive.

The IFT particle can be fractionated into two complexes termed IFT-A (made of 6 proteins, required for retrograde transport) and IFT-B (made of at least 16 proteins, essential for anterograde transport)^22^. The IFT-B complex can be further sub-divided into a 10 subunit IFT-B1 and a 6 subunit (IFT172, IFT80, IFT57, IFT54, IFT38 and IFT20) IFT-B2 complex^23^. IFT172 is the largest of the IFT proteins with a molecular weight of 200 kDa and belongs to the IFT-B2 complex^22–24^. Genetic studies showed that IFT172 is required for ciliogenesis in *Chlamydonomas*^25^, *Trypanosoma brucei*^26^, *Tetrahymena*^27,28^, *D.melanogaster* neurons^27^ and for cilium-mediated hedgehog signaling in mouse embryos^29^, and null alleles of *IFT172* in mice are embryonic lethal. Mutations in the human *IFT172* gene result in skeletal ciliopathies^30^. Endogenous IFT172 is found to localize to punctate foci clustering at the base of the cilium^30^ as well as at the tip of the cilium, where it may be involved in IFT-train tip-turnaround via an unknown mechanism^31^. IFT172 is incorporated into the IFT complex through a salt-labile interaction with the calponin homology (CH) domain of IFT57^23^. The predicted domain organization of IFT172 is structurally similar to COP-I,II and clathrin proteins, however, the biochemical behavior of IFT172 is not well understood.

In this report, we hypothesize that IFT172 may interact with membranes, and characterize the molecular properties of purified *Chlamydomonas* IFT172 in membrane binding. Recombinantly prepared IFT172 shows an architecture of two ~10 nm-sized globular domains followed by a rod-like extension, which is consistent with the bioinformatics prediction of two β-propeller domains appended by a long stretch of alpha-solenoids. Interestingly, IFT172 can also employ a second distinct conformation, with a square shaped organization (closed conformation). Furthermore, using giant unilamellar vesicles (GUVs) and cryo-EM, we find that IFT172 interacts with membrane surfaces and surprisingly remodels these membranes into small entities of ~20 nm in size. The pinching of the vesicle surface happens with as little as 50 nM IFT172 by membrane surface clustering, showing the high specificity of the protein for membranes. Larger foci formation of IFT172 can be observed at the initiation stage of ciliogenesis, implying the IFT172-lipids association has a role during the cilia formation.

## Results

### IFT172 readily associates with lipids

**Fig. 1:**
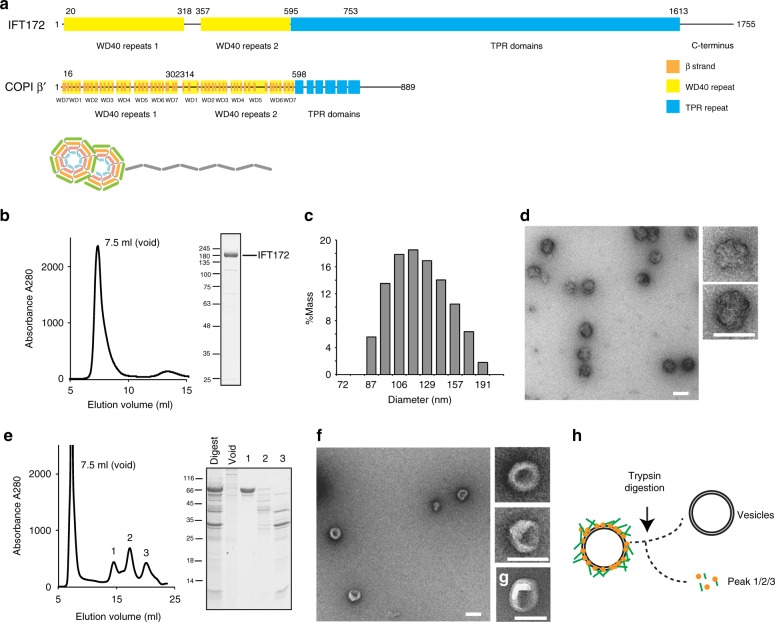
Characterization of recombinant *Chlamydomonas* IFT172. **a** Predicted domain architecture of IFT172 from *Chlamydomonas*. The predicted WD40 repeats (in yellow) and TPR (tetratricopeptide repeat) domain (in blue) were defined by HHpred. IFT172 shows a similar domain organization to COPI/II and clathrin. COPI β’ from *Saccharomyces cerevisiae* is shown for comparison. **b** Size-exclusion chromatography (SEC) profile of recombinant full-length IFT172 and Coomassie-stained SDS-PAGE analysis of recombinant IFT172 after size-exclusion chromatography. **c** Dynamic light scattering (DLS) measurement of IFT172 shows a uniform particle size distribution of IFT172 with an average diameter of ~120 nm with 100% mass. **d** Negative stain EM micrograph of IFT172 showing globular oligomer formation. Scale bar: 100 nm. **e** SEC profile of IFT172 proteolyzed products after limited-proteolysis (left) and SDS-PAGE of the peak fractions from SEC (right). **f** Negative stain EM micrograph of the fraction from the void peak in **e** shows round particles with smooth surface. Scale bar: 100 nm. **g** Negative stain EM micrograph of the liposomes from Folch fraction I as a control. Scale bar: 100 nm. **h** Scheme describing the experimental procedure of **e** and **b**

IFT172 has a molecular weight of ~200 kDa, and is the largest component of the IFT complex^22,24^. Bioinformatics analyses using HHpred/Phyre2^32,33^ predicted that IFT172 has two N-terminal β-propellers (~65 kDa) followed by a ~1100 residues α-solenoid (~115 kDa), with significant sequence similarity to known vesicle coatomer proteins such as COPI/II and clathrin subunits (Fig. 1a). To characterize the behavior of IFT172 in vitro, we recombinantly prepared *Chlamydomonas* IFT172 using the eukaryotic insect cell expression system. IFT172 expressed with a C-terminal His-tag appeared in the soluble fraction after centrifugation of the cell lysate, but the elution from Ni-NTA beads resulted in a turbid white solution indicative of a high lipid content (Supplementary Table1). While we have previously used purified IFT172^23^ that eluted at an estimated molecular weight of around 400 kDa in size exclusion chromatography (SEC), a significant fraction of IFT172 eluted in the void volume suggesting a molecular weight of more than 1 M Da (Fig. 1b). Although elution at the void volume is normally a sign of protein aggregation, dynamic light scattering (DLS) analysis showed a uniform particle size distribution of IFT172 with an average diameter of ~120 nm suggesting an oligomeric assembly of the protein (Fig. 1c). To further characterize the SEC-purified IFT172 lipid-associated mixture, negative-stain electron microscopy (EM) was performed, revealing rather uniform, large, globular objects with a size of ~75 nm and a rough surface (Fig. 1d). These were composed of IFT172 and various small components with molecular weights in the range of lipids according to mass spectrometric analysis (Supplementary Table1). Based on this, we hypothesized that these oligomers may be composed of a lipid-based vesicle-core covered with IFT172 protein. To test this hypothesis, the IFT172 oligomers were treated with the protease trypsin, and SEC was carried out to separate proteolyzed products (Fig. 1e–h). SEC showed new appearances of proteolyzed protein species (Fig. 1e, left, labeled 1–3) in addition to the original void signal, which no longer contained significant amounts of IFT172 (Fig. 1e, right). The EM observation of the void product showed smooth, round particles (Fig. 1f), typical of uncoated liposomes (Fig. 1g). These data support the notion that the purified particles represent lipid vesicles covered by IFT172 protein and not soluble aggregates.

### IFT172 adopts distinct closed and open conformations

**Fig. 2:**
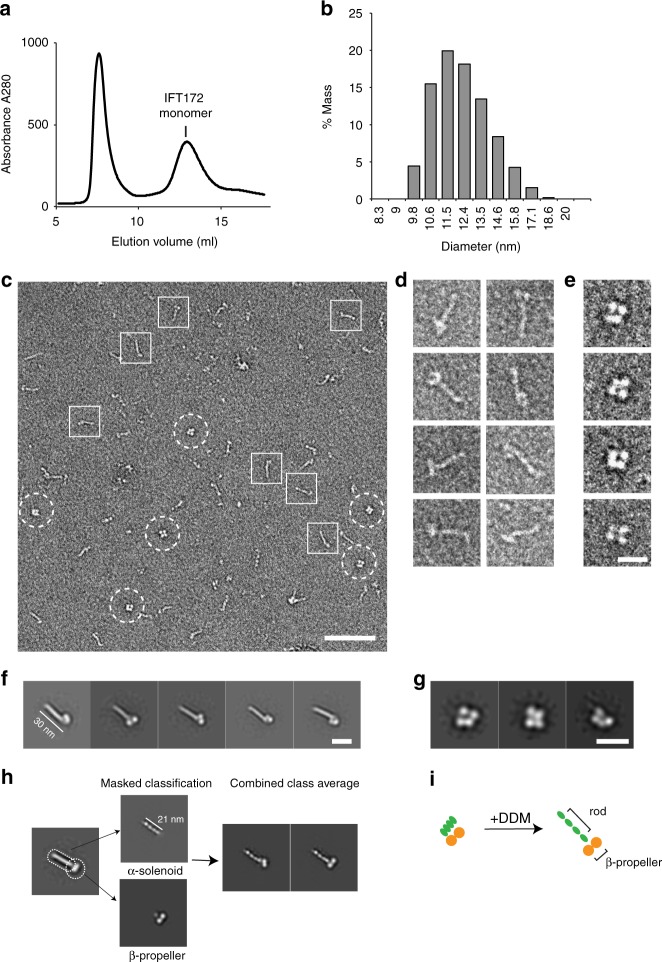
Monomeric architecture of recombinant *Chlamydomonas* IFT172. **a** SEC profile of recombinant full-length IFT172 with 0.1% DDM shows an increased amount of the monomer peak. **b** DLS measurement of IFT172 monomer shows the size of the particle in the monomer peak is around 11 nm. **c** Negative stain EM micrograph of monomeric IFT172 with 0.1% DDM, showing an open (square) and a closed (circle) conformation. Scale bar: 100 nm. **d**, **e** Representative IFT172 monomers in an open (**d**) and closed (**e**) conformation. Scale bar: 20 nm. **f**, **g**. Reference-free 2D class averages of IFT172 in an open (**f**) and a closed (**g**) conformation. Scale bar: 20 nm. The open conformation shows a rod-like structure with 30 nm in length, while the closed IFT172 contains 4 sub-domains. **h** Detailed 2D averages of IFT172 by domain masking and assembly strategy. Scale bar: 20 nm. The original average views are shown on the left side. To obtain detailed features for each domain, soft masks (dotted lines) were applied to facilitate an individual alignment of the head domain and tail domain. Two complete IFT172 molecular are generated by aligning the two components. The N-terminal head region shows two globular densities with a size of ~5 nm each. The rod part shows 4 globular domains with 4 nm each, which could be the features by TPR alpha solenoid motifs. **i** Schematic representation of the conformational change of monomeric IFT172 induced by DDM

To isolate monomeric IFT172 from the lipid containing IFT172 oligomers, the detergent n-dodecyl β-D-maltoside (DDM) was added to solubilize IFT172. Upon the addition of DDM, the fraction of the large IFT172 oligomers was lowered, and we observed a concomitant increase of a peak for isolated monomeric IFT172 with an estimated size by SEC of ~450 kDa (Fig. 2a). This corresponding fraction showed the size of ~11 nm by DLS (Fig. 2b). To visualize the domain architecture of IFT172, negative-stain EM of this detergent purified IFT172 was carried out (Fig. 2c). The purified IFT172 showed two different morphologies, 1) a 30-nm long rod-like architecture (rod), with a ~10 nm globular attachment (head) (Fig. 2c, square, and Fig. 2d), and 2) a globular architecture (Fig. 2c, circle, and Fig. 2e). The rod-like architecture is largely consistent with the predicted domain organization of the IFT172 (Fig. 1a). 2d averages focusing on the head domain (Fig. 2f–h) yielded more defined features, revealing two individual ~5 nm, globular densities, likely corresponding to the two β-propeller domains. Surprisingly, the density of the rod domain was subdivided into four small globular domains with a size of 4 nm each (Fig. 2g). The rod domain is comprised of alpha-solenoidal TPR motifs predicted from sequence analysis (Fig. 1a), but the observed sub-domain organization has been unknown so far. On the other hand, 2D averages of the compact IFT172 revealed a ~12 nm globular structure with 4 subdomains inside (Fig. 2h). Presumably, two of these subdomains correspond to the WD40 β-propeller domains, and the rod domain might be closed up by connecting to the head domains (Fig. 2i).

To avoid the influence of the residual detergent during the detergent-dependent IFT172 purification, we revised the purification by employing Q-Sepharose anion-exchange chromatography without using detergent. Interestingly, the purified IFT172 without detergent only contained IFT172 with the closed conformation (Supplementary Figure 1A), indicating that DDM facilitates the opening of IFT172. We then added DDM to the purified, globular IFT172, confirming that IFT172 converts into the open conformation, showing that IFT172 can employ both closed and open conformations (Supplementary Figure. 1A-C).

### IFT172 interacts directly with and vesiculates membranes

**Fig. 3:**
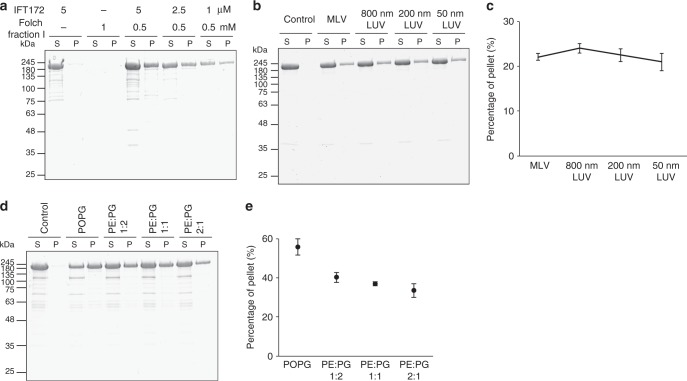
Liposome sedimentation assays of *Chlamydomonas* IFT172. **a** Co-sedimentation assay of IFT172 with Folch fraction I multilamellar vesicles (MLV), showing direct binding of IFT172 to membrane. S: supernatant and P: pellet. **b**, **c** Co-sedimentation assay of IFT172 with Folch fraction I MLV and large unilamellar vesicles (LUVs) of different sizes (**b**) and their quantification (**c**). Error bars in (**c**) depict standard deviations from three separate experiments. IFT172 has no preference for different membrane curvatures. **d**, **e** Co-sedimentation assay of IFT172 with liposomes containing different ratios of POPE and POPG (D) and their quantifications (**e**). Error bars in **e** depict standard deviations from three separate experiments. IFT172 has a preference for negatively charge lipids. The experiments were performed three independent times

**Fig. 4:**
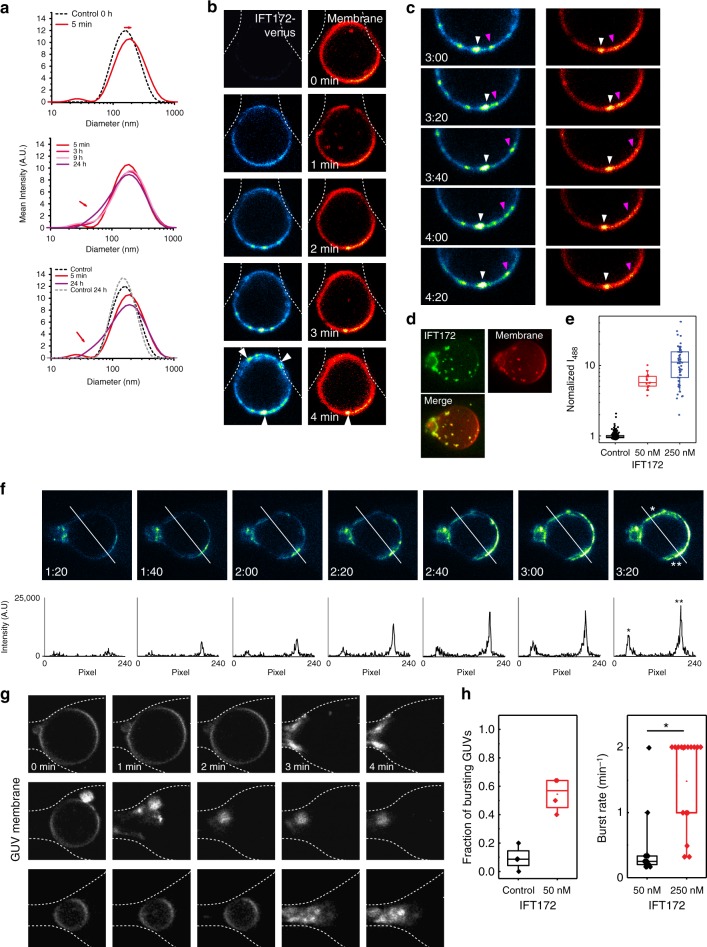
IFT172 binds to and destabilizes LUVs/GUVs. **a** DLS measurement of IFT172 with 200 nm LUVs. Dotted black line: control t=0, dotted gray line: control t=24 h. Colored lines follow with 5 min (red), 3 (dark pink), 9 (light pink) and 24 h (purple) incubations. **b** IFT172-venus binds to GUVs (DOPE-ATTO655) and forms clusters on membrane surfaces (arrowheads). IFT172 (50 nM) was added to GUVs and imaged at 20 s intervals. ATTO655 shown in Red Hot, IFT172-venus in Green Fire Blue color-code from FIJI. Dotted lines outline the micro-fabricated trap. **c** Tracking of clusters on the GUV shown in **b**. Snapshots are taken at 20 s intervals. Two examples of cluster movements are indicated with magenta and white arrowheads. **d** Z-projection of an entire GUV, visualizing scattered clusters on the vesicle surface. **e** Intensity of IFT172 on GUVs. Raw data points are shown in scattered dot plot. *n* = 273 for control, *n* = 17 for 50 nM IFT172 and *n* = 55 for 250 nM IFT172. The experiments are repeated at least three times. **f** IFT172 on a GUV surface and the line plots of intensity of IFT172 at different time points. The line plot shows that the intensity of IFT172 clusters (right peak) grew faster than the background IFT172 binding (left peak). **g** Collapse of the GUVs (gray) via binding of IFT172. IFT172 (50 nM) was added to GUVs, and imaged in one minute intervals. **h** Statistics of GUV collapse after IFT172 binding. For 250 nM IFT172 box plot, the median is identical to the upper end of the box. Burst rates of vesicles were analyzed with Mann–Whitney *U* test (*z* = −4.34, p < 1.5 × 10^−5^). See Source Data. These experiments were done 3 independent times (17 data points). For box plots (E and H), boxes denote the 25th and 75th percentile, whiskers denote the 10th and 90th percentile, squares denote the mean, and the mid line the median

On the basis of the observation that IFT172 co-purifies with lipids, we hypothesized that IFT172 may interact directly with membranes. To test this hypothesis, detergent-free monomeric IFT172 was mixed with liposomes (Folch fraction I) and protein-liposome co-sedimentation assays were performed. While IFT172 alone did not sediment, a substantial amount of IFT172 (up to 40%) was co-sedimented in the presence of liposomes (Fig. 3a). IFT172 did not show any apparent preference to different sizes of vesicles (Fig. 3b, c), indicating that membrane curvature is not a critical factor for the binding of IFT172 to membrane surfaces. We rather observed a preference of IFT172 for negatively charged lipids (Fig. 3d, e). As such, going forward we used Folch Fraction I for further membrane experiments. Further, using analytical DLS, the interaction of IFT172 with membrane was tested. Immediately after adding 500 nM IFT172, we observed that the average size of liposomes of 200 nm in diameter increased by 33%, presumably due to the binding of the protein to the liposome surface (Fig. 4a, top). Then, the peak position of vesicles gradually shifted to smaller sizes, while the size distribution became broader (Fig. 4a, middle and bottom). This change of the vesicle size suggests remodeling of liposomes in the presence of IFT172. Using GUVs and fluorescently labeled lipids, the interaction of Venus-tagged IFT172 with membranes was directly followed (Fig. 4b). Supporting the DLS observation, an association of the IFT172-Venus with GUVs was observed (Fig. 4b–e). To follow the dynamics of the interaction, real-time recordings of GUVs were performed (Fig. 4b, c, Supplementary Movie 1). IFT172-Venus started to cover the surface of a GUV immediately after addition and the intensity of decorated IFT172 maximized within ~2–4 min. When the IFT172 concentration was at its detection limit (50 nM), IFT172 displayed an uneven coverage of GUVs (Fig. 4b-c, arrowheads and 4D), indicating a clustering of the protein on the membrane surface. Interestingly, sometimes co-clustering of lipids to IFT172 was observed on GUVs, while the membrane itself typically shows a smooth surface without IFT172 (Fig. 4b, 0 min). This means that the membrane remodeling was accompanied with IFT172 clustering. These clusters were mobile (Fig. 4c), suggesting that the IFT172-membrane clusters were still attached to the GUV surface, as the lipids within vesicles are fluid. The clustering behavior of IFT172 is likely due to the association of IFT172 to the patches of negatively charged areas within GUVs, judging from the finding that IFT172 prefers negative charged lipids (Fig. 3d). The intensities of the covered proteins grew in a temporal manner and particularly, the growth was faster at IFT172 cluster sites compared to the rest of the GUV. (Fig. 4f). FRAP (Fluorescence recovery after photobleaching) experiments showed a rapid recovery of IFT172 signal after photobleaching, suggesting that the IFT172 coat on membrane surfaces is mobile as well (Supplementary Figure 2). Interestingly, we observed that ~60% of GUVs collapsed within 10 min after adding IFT172 (Fig. 4g, h left). Some of the GUVs that remodeled into smaller entities were trapped on the supporting chip (Fig. 4g, 4 min). The frequency of GUV collapse depended on the concentration of added IFT172, as the frequency increased by a factor of 10 upon addition of 250 nM IFT172 (Fig. 4h right). The critical concentration for membrane remodeling appears to be around 10 nM under the given experimental condition, as collapsing vesicles were no longer observed below this concentration. These results show that IFT172 interacts with membranes, clusters on the membrane surface, and remodels the membrane.

**Fig. 5:**
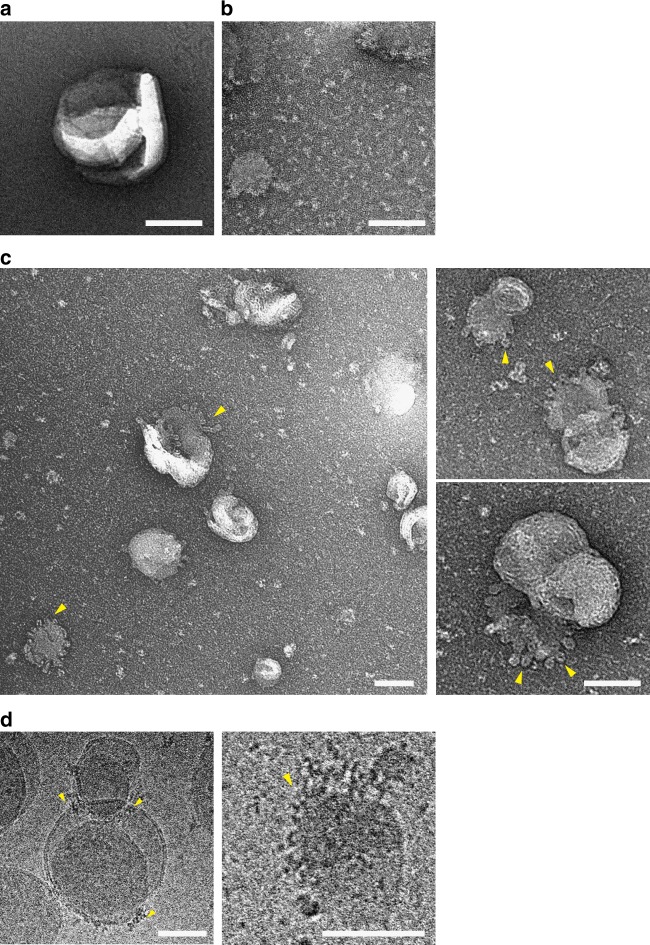
Electron-microscopic visualization of IFT172 with Folch fraction liposomes. **a** Negative-stain EM image of Folch fraction I liposomes as control. Scale bar: 100 nm **b** Negative-stain EM image of IFT172 (1 µM) with Folch fraction I liposomes (0.2 mM). Scale bar: 100 nm. The membrane became deformed into small pieces. **c** Negative-stain EM images of IFT172 (250 nM) with Folch fraction I liposomes (0.2 mM) showing the remodeling of the membrane surface by IFT172. Scale bar: 100 nm. Small (~20 nm) vesicles (yellow arrowheads) get pinched off from the membrane surface. **d** Cryo-EM images of IFT172 (500 nM) with Folch fraction I liposomes (0.4 mM). Scale bar: 100 nm

To more closely examine the remodeling events of vesicles by IFT172, the mixture of IFT172 with liposomes was observed by negative-stain EM. Compared to intact liposomes (Fig. 5a), the addition of 1 µM IFT172 to 200-nm liposome resulted in fragmentation of the liposomes (Fig. 5b). Lowering the protein concentration to 250 nM facilitated a better visualization of the process, showing the striking association of IFT172 and the formation of relatively uniform, ~18 nm ring-like structures ‘budding out’ from liposomes (Fig. 5c, d, arrowheads). Next, the IFT172-liposome mixture was fractionated by SEC (Supplementary Figure 3A). The fraction of ring-like structures (Supplementary Figure 3B) shifted to longer elution times and mass spectrometry confirmed that it contained both lipids and IFT172. (Supplementary Figure 3C and D)

### IFT172 N-terminal β-propeller region binds to membrane

**Fig. 6:**
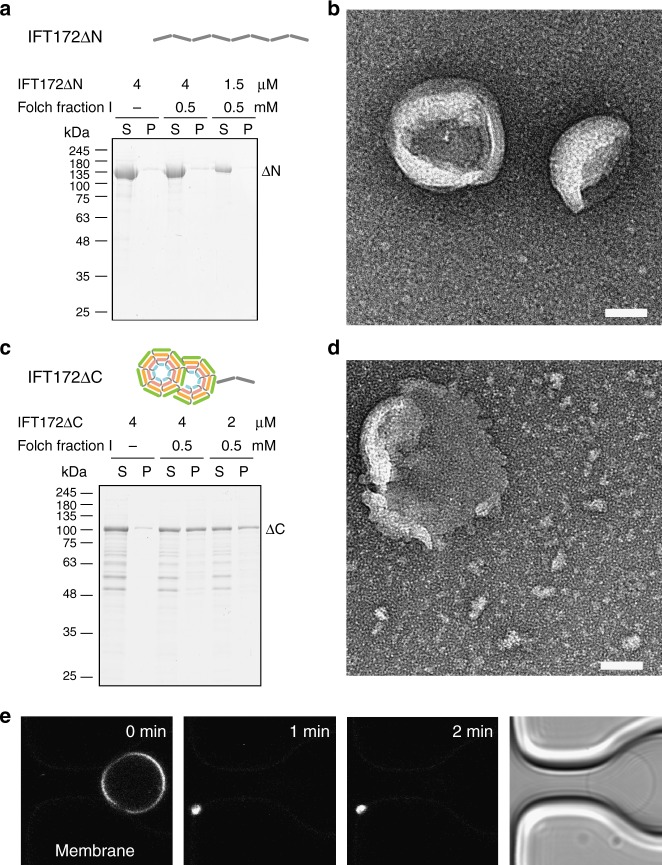
Mapping of membrane-binding region in IFT172. **a** Co-sedimentation assay of IFT172ΔN with Folch fraction I MLV. IFT172ΔN shows no affinity to membranes. **b** Negative-stain EM images of IFT172ΔN with Folch fraction I liposomes do not show remodeling of the membrane surface. Scale bar: 50 nm **c** Co-sedimentation assay of IFT172ΔC with Folch fraction I MLV. IFT172ΔC shows affinity to membranes. **d** Negative-stain EM images of IFT172ΔC with Folch fraction I liposomes show the remodeling of the membrane surface. Scale bar: 50 nm. **e** Observation of the effect of IFT172ΔC on GUVs. GUVs collapse after IFT172ΔC addition

To assess which domain of IFT172 is involved in membrane binding, two fragments lacking either the N-terminal globular domain (residues 590-1755, IFT172ΔN), or lacking most of the C-terminal rod domain (residues 1-968, IFT172ΔC) (Fig. 6) were prepared and tested for liposome interaction. A co-sedimentation assay of IFT172ΔN with liposomes showed that the protein does not bind to membranes (Fig. 6a), and no apparent remodeling of membrane surface was observed by negative stain EM (Fig. 6b). On the other hand, IFT172ΔC showed interactions with liposomes in a co-sedimentation assay (Fig. 6c). The breakage of GUVs was also observed after addition of IFT172ΔC in the GUV assay (Fig. 6e), similar to what we showed using full-length IFT172. Negative-stain EM revealed deformation of liposomes after mixing with IFT172ΔC (Fig. 6d). These results together indicate that the association of IFT172 with membranes is mediated by the N-terminal β-propeller.

### IFT57 competes with lipids for the binding to IFT172

**Fig. 7:**
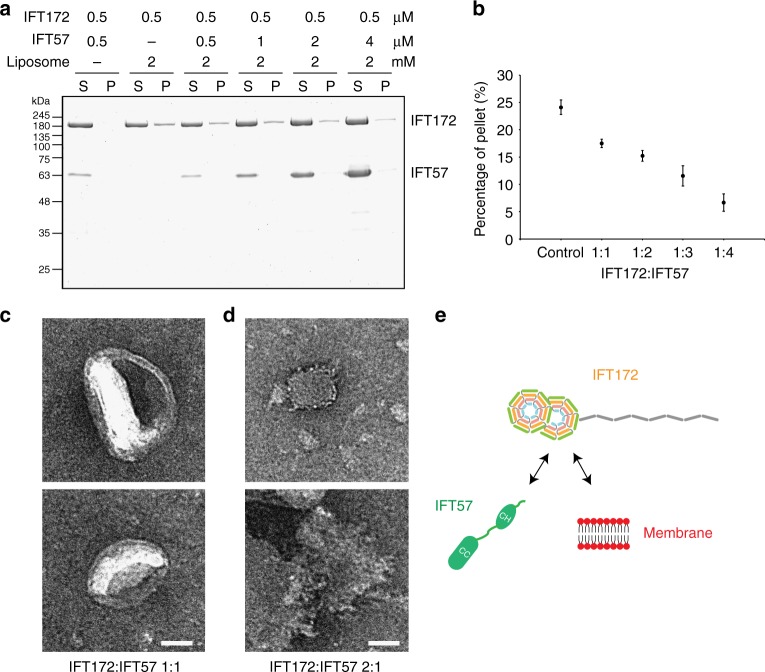
IFT57 competes with membrane for binding to IFT172. **a**, **b** Co-sedimentation assay of IFT172 with Folch fraction I MLV in presence of IFT57. The quantification (**b**) shows the decrease of the amount of IFT172 in the pellet in the presence of IFT57. Error bars depict standard deviations from three separate experiments. These experiments were performed three independent times. **c** Negative-stain EM images of the IFT172/IFT57 dimer in 1:1 molar ratio with Folch fraction I liposomes show no remodeling of the membrane surface. Scale bar: 50 nm. **d** Negative-stain EM images of IFT172/IFT57 dimer in 2:1 molar ratio with Folch fraction I liposomes show the remodeling of the membrane surface. Scale bar: 50 nm. **e** Schematic representation of IFT172 interacting with IFT57 or membranes

IFT57 is reported to be a direct interaction partner of IFT172 within the IFT-B2 complex, and a previous analysis showed that this interaction is mediated by the IFT172 N-terminal region and the IFT57 Calponin Homology (CH) domain^23^. We therefore tested the binding of IFT57 to IFT172 in the presence of membranes. The liposome co-sedimentation assay showed that IFT57 has no interaction with membranes (Fig. 7a). As more IFT57 is added to the IFT172-liposome mixture, the amount of pelleted IFT172 decreased, showing that less IFT172 interacts with membrane surfaces in the presence of IFT57 (Fig. 7b). Notably, IFT57 does not co-sediment in any of these conditions, indicating that the IFT57-IFT172 complex does not interact with liposomes.

### IFT172 is fractionated as a membrane-associated protein

To characterize the cellular localization of IFT172, we performed immunofluorescence microscopy using RPE-1 cells (human retinal pigment epithelium cells) stained for endogenously expressed IFT172 (Supplementary Figure 4A and 4B). Upon starvation for 24 h, which facilitates ciliogenesis, IFT172 showed co-localization with the membrane-associated ciliary marker Arl13b (ADP-ribosylation factor-like 13b) along the length of the cilium (Fig. 3c)^34^, in agreement with a previous report^30^ also validating the specificity of the antibody. IFT172 formed intensive localizations at the base and tip of a cilium (Supplementary Figure 4A-C) but no apparent specific colocalization of IFT172 to large cellular components was found. Therefore, we further performed a centrifugation-based cellular fractionation procedure^35^ to analyze the sub-cellular localization of IFT172 in RPE-1 cells. IFT172 was found in the lipid-rich organelle fraction, which suggests that IFT172 is associated to lipid without activation, but too small to be visualized under tested fluorescence microscopic setups (Supplementary Figure. 4C).

## Discussion

Possible biological functions of IFT172 are Golgi-to-cilium transport of vesicles^36^, endo-/exocytosis at the ciliary pocket^37^, involvement in train formation^38^, participation in cell division at the cleavage furrow^17^ and the IFT train turnaround at the ciliary tip^39^. Many of these suggested functions of IFT172 require an interaction of the protein with the membrane surface. Here, we report that IFT172 clusters on membrane surfaces in vitro at a concentration of as little as 50 nM, showing a strong affinity to membranes, and mapped the membrane-interacting region to the N-terminal domain of IFT172. The β-propeller domains of IFT172 have a negatively charged surface at the inner lumen of the β-propeller 1 according to homology modeling, similar to the charge distribution on the surface of beta COPI^40^. The COPI complex contains a direct membrane recruiting subunit (Arf1)^41^, but the charged surface of beta COPI is also located proximal to the membrane. Together, this suggests that IFT172 might recognize membrane surfaces through its β-propeller blade surface. Other than the charged blade, IFT172 does not have an obvious membrane binding or curving domain. However, it was recently reported that membrane curvature formation resulting in a fission event could occur due to the crowding of proteins^42^. It is possible that membrane remodeling by IFT172 is induced by local crowding causing steric pressure to the membrane surface, which might be facilitated by self-association of IFT172 on a negatively charged surface of the membrane, as we observed preferential binding of IFT172 to this class of lipids (Fig. 3d, e).

Archetypical vesicle coat proteins such as clathrin or COPI/II proteins^43–46^ form well-ordered coatomers with internal symmetry^46^. Previous studies highlighted the similarities between IFT172 and proteins that are involved in coatomer formation^47^. Our homology detection using HHpred^33^ also indicated that clathrin has significant similarity (E-value of 10^−29^) to IFT172. Purified IFT172 showed a striking and fast interaction with vesicles and also remodels membrane surfaces enough to pinch off small vesicle-like structures. Furthermore we showed that the interaction of IFT172 with membranes occurs via the β-propeller domain, resembling the proximal location of the COPI β-propeller domain to the membrane surface of COPI-vesicles^40^. Taken together, we speculate that IFT172 interacts with liposomes, possibly to pinch off a small lipid fracture, or even to form a well-ordered coat in the presence of other still unknown interaction partners.

In our search for the binding partners that possibly facilitate an organized coat-like structure, our primary choice was the known IFT172 binding partner IFT57, another component of the IFT-B2 complex^23^. Interestingly however, we observed a competitive effect of IFT57 towards the membrane binding of IFT172. It is interesting to note that IFT172 may indeed be a peripheral component of the IFT complex as described^22^ and that it may alternate between the IFT-B2 and membrane. In this sense, IFT172 might be a modulated cargo adapter that is carried by IFT, or the glue that is first anchored to the membrane surface at the base of cilia, and then, recruits other components for IFT train assembly.

What are the mechanisms for switching between anterograde and retrograde IFT at the ciliary tip allowing for a full transport cycle? When kinesin II-driven anterograde IFT trains reach the distal end of the axoneme, they are rapidly converted into dynein 2-driven retrograde IFT trains through poorly understood mechanisms. Photobleaching experiments revealed that this process takes less than 3 s in *Trypanosomes*^48^. In *C.elegans*, single-molecule fluorescence microscopy revealed that IFT-A particles turn around quickly at the ciliary tip (600 ms) whereas IFT-B particles linger longer as they reside at the tip for an average of 3 s before returning to the base by retrograde IFT^49^. IFT172 was shown to be required for tip-turnaround in *Chlamydomonas*, as a temperature sensitive C-terminal point-mutant of IFT172 accumulates IFT proteins at the ciliary tip^39^. Consistently, it is known that IFT172 truncations lacking parts of the alpha-solenoid at the C-terminus are sufficient to support anterograde IFT in *Tetrahymena*, but accumulate at the ciliary tip together with other IFT proteins indicative of a tip-turnaround defect^28^. It thus appears that the N-terminal β-propeller domain of IFT172 is involved in IFT train formation and anterograde transport while the C-terminal domains are required at the ciliary tip to switch to retrograde transport. Our in vitro observation showed that the N-terminal β-propeller has a mutually exclusive interaction with IFT57 and membrane. This may indicate that the membrane-binding module of IFT172 is inhibited when it is incooporated in the IFT complex to form an IFT train. During the IFT turnaround, disassembly and reconfiguration of IFT trains, IFT172 may park at the membrane surface by associating with the membrane at the tip of cilia. On the other hand, the alpha-solenoid rod domain may play an additional role in re-association of IFT172 to the rest of IFT complex^28^. Moreover, the tip of Chlamydomonas flagella is characterized by a flagellar tip complex (FTC), which links the MT singlets of the axoneme to the overlaying flagellar membrane^50^. The IFT172 interacting protein EB1 was shown to reside at the FTC^51^ and may regulate IFT172 disassembly from the IFT complex and/or membrane association by IFT172. The exact molecular mechanisms of tip turn-around remain to be elucidated.

## Methods

### Protein expression and purification

The coding sequence of IFT172, IFT172ΔN (residues 590-1755) and IFT172ΔC (residues 1-968) from *Chlamydomonas reinhardii* were gene synthesized, or sub-cloned into multiple cloning site 2 (MCS2) of pFL vector, and a His-tag (6 × HIS) was added to the C-terminus of IFT172 by PCR. Primers used for this study are listed in supplementary table2. Then DH10 BAC cells (Thermo Fisher Scientific) were transformed with the plasmid for making recombinant production of baculoviral DNA. Sf9 insect cells (Sigma-Aldrich) were transfected with recombinant viral DNA for making recombinant baculoviruses. HighFive cells (Invitrogen) (200 ml, 1 × 10^6^ cells/ml) were infected with IFT172-His tag virus and incubated at 26 °C for 3 days. The cells were harvested by centrifugation (1000 × *g*, 15 min), and resuspended in 5 times lysis buffer (20 mM Hepes pH 7.4, 10 mM KCl, 1.5 mM MgCl_2_, 250 mM sucrose) with a protease inhibitor tablet (complete tablet, EDTA-free; Roche) and 5 mM β-mercaptoethanol. Cells were transferred to a Dounce homogenizer and homogenized around 20 strokes, and it was left on ice for 20 min for complete break up. The crude cell lysate was centrifuged at 750 xg for 10 min to pellet the nuclei. NaCl was then added to the supernatant to a final concentration of 200 mM, followed by further centrifugation (10,000 xg, 30 min, 10 °C). His60 Ni Resin (Clontech) was used for affinity binding, and the Ni Resin was washed with 20 mM Hepes pH 7.4, 150 mM NaCl, 1 mM DTT. The protein was eluted with 20 mM Hepes pH 7.4, 150 mM NaCl, 300 mM imidazole and 1 mM DTT. The eluted IFT172 was concentrated and loaded onto a Superose 6 10/300 (GE Healthcare) size-exclusion chromatography column equilibrated with 20 mM Hepes pH 7.4, 150 mM NaCl, and 1 mM DTT. To further investigate the IFT172 association with lipids, vesicle fractions were purified by ultracentrifugation starting from insect cell material over-expressing IFT172. The results of this procedure showed that IFT172 co-purified in the lipid-fraction after the last centrifugation step at 100.000 xg, 30 min.

To purify the IFT172 monomer, the same purification procedure was used except that 0.1% DDM was added to all the buffers. To purify the detergent-free IFT172 monomer and IFT172-venus for membrane-binding assays, the elution from His60 Ni Resin was then loaded onto anion-exchange column (5 ml HiTrapQ-Sepharose, GE Healthcare) before size-exclusion chromatography and DDM was omitted in the purification last steps.

Purification of IFT57 was carried out using bacteria expression system^23^. Briefly, full-length *Chlamydomonas* IFT57 was cloned into pEC vector with N-terminal cleavable His tag and expressed in E. coil BL21 (DE3) (New England Biolabs). The bacterial culture was grown in TB medium at 37 °C until O.D. (600 nm) of around 2, then the temperature was reduced to 18 °C and 0.5 mM IPTG was added for over-expression overnight. Cells were harvested and lysed in 50 mM Tris-HCl pH 7.5, 150 mM NaCl, 10% glycerol, 5 mM β-mercaptoethanol, 1 mM PMSF, 25 μg/ml DNase I. The soluble fraction was loaded on a Ni^2+^ -NTA column (5 ml, Roche). The column was washed with lysis buffer with additional 1 M NaCl and the protein was eluted with lysis buffer contains 500 mM imidazole. The eluted fraction was dialyzed against 20 mM Tris-HCl pH 7.5, 50 mM NaCl, 10% glycerol, 5 mM β-mercaptoethanol with TEV protease to remove the His tag. The dialysis product was further purified by an ion-exchange column (anion exchanger, 5 ml HiTrapQ-Sepharose, GE Healthcare) to remove contamination and cleavage tags. Size exclusion chromatography (SEC) was finally applied with 10 mM Hepes pH 7.5, 150 mM NaCl, 1 mM DTT.

### Limited proteolysis

Limited proteolysis of IFT172-lipid oligomers was performed in 20 mM Hepes pH 7.4, 150 mM NaCl, and 1 mM DTT. 0.05 mg/ml final concentration of Trypsin was incubated with IFT172-lipid oligomer at room temperature for 2 h. Samples were analyzed by SDS-PAGE and size-exclusion chromatography on Superose 6 10/300 (GE Healthcare) column.

### Liposomes preparation

Liposomes were prepared from a bovine brain extract, Folch Fraction I (Sigma Aldrich) by evaporating the chloroform of the lipid under a nitrogen-stream and further incubation under vacuum. Multi-lamellar vesicles (MLVs) were generated by hydrating the dried lipid with 20 mM Hepes, 150 mM NaCl, pH 7.5 to a concentration of 2 mg/ml. Large unilamellar vesicles were generated by extruding the MLV-suspension through filter (Avanti) with pore sizes of 200 nm, 100 nm, and 50 nm.

GUVs were generated using the gentle hydration method^52^. Briefly, 100 µl of 10 mg/ml Folch Fraction I with 0.1% ATTO 655 labeled DOPE (ATTO-TEC) dissolved in 20:9:1 chloroform: methanol: H_2_O was dried on glass test tube under a nitrogen-stream and subsequent incubation under vacuum, then 1 ml of 10 mM Hepes, 240 mM sucrose, pH 7.5 was added gently to the glass tube without disturbing the lipid layers. The tube was incubated at 75 °C overnight. The bulky cloud floating in the middle of the solution, containing the GUVs, was transferred to 10 mM Hepes, 150 mM NaCl, pH 7.5 for fluorescence imaging.

### Liposomes sedimentation assay

IFT172 or IFT172/57 in 20 mM Hepes, 150 mM NaCl, pH 7.5 was incubated with MLVs or large unilamellar vesicles for 15 min on ice. Soluble proteins were separated from liposome-bound proteins by ultracentrifugation for 20 min at 135,000 xg using a TLA100.3 rotor in a TL-100 tabletop ultracentrifuge (Beckman). Both supernatant and pellet fractions were collected and analyzed using SDS-PAGE.

### Negative stain EM

To observe IFT172 in lipid binding form and monomer form from SEC, samples (0.05 μM) were applied to homemade carbon grids. For the observations of the change of liposomes by IFT172, IFT172ΔC and IFT172ΔN, 0.25 uM proteins were mixed with 0.2 mM MLV or 200 nm LUV, grids were made without dilution. The grids were staining with 1% (w/v) uranyl-acetate staining and images were recorded on a CM200 (FEI, 160 kV) under low-dose mode with magnification of 50,000 corresponding to 2.16 Å/pixel. For IFT172 monomer architecture analysis, micrographs were recorded with magnification of 38,000x corresponding to 2.78 Å/pixel. e2boxer.py from EMAN2 package^53^ for semi-automatically particle picking and two-dimensional (2D) reference-free classification was performed using the RELION software package^54^. To obtain the domain architecture of open conformation of IFT172, about 6000 particles were picked and applied for domain masking and an assembly strategy. Soft masks for each domain were generated by Fiji. After drawing the domain shapes by the ‘wand tool’ in Fiji^55^, the masks were soft-edged by enlarging ~9 pixels and filtering with Guassian blur in Fiji. Masks for N-terminal β-propeller globular domain of IFT172 and the C-terminal alpha-solenoid rod were applied to individual, aligned single particles images using ‘bmask’ from BSOFT software package^56^, and the resulting images for each domain were further aligned and average using Relion, and the 2D averages were combined with ‘badd’ from BSOFT. Observation of the IFT172/IFT57 complex was attempted using negative stain EM, however the complex fell apart on an EM grid, making it unfeasible for the interpretation of the morphology of the complex.

### Cryo-EM

For the observation of IFT172 interaction with membrane under cryo-EM, IFT172 (0.5 μM) and LUVs in 200 nm (0.4 mM) were mixed and vitrified immediately. A concentration of 4 μl sample without dilution was placed onto a glow-discharged Quantifoil grids (Cu, R2/2) and then blotted for 6 s with blot force 6 to remove the excess solution with Vitrobot Mark IV (FEI). The Vitrobot chamber was operated at 4 °C with 100% humidity. The grids were observed on a Tecnai F20 (FEI) with an acceleration voltage of 200 kV with a nominal magnification of ×29,000 corresponding to a pixel size 3.72 Å/pixel. The micrographs were taken with a CCD camera (FEI, Eagle) with a defocus range of −2 to −4 μm.

### Microfluidic chip fabrication and preparation

PDMS microfluidic chips with vesicle traps were fabricated using soft lithography. The vesicle trap geometry was adapted from ref^57^. First, we increased the trap density to facilitate time-lapse imaging of multiple vesicles in a single field of view. We then used a layered arrangement of progressively narrower trap posts. The first quadrants had 8 µm spacing between trap posts, with subsequent quadrants narrowing to nominally 6, 4.5, and 3 µm gaps between both trap posts. In this arrangement, smaller vesicles that could not be captured in the wide traps were trapped in the narrower traps downstream. SU8 master moulds of 10 and 20 µm were produced on a 4 inch silicon wafer (University Wafer) using SU-8 2010 or 2015 (Microchem corp.), according to the manufacturers data sheet and developed using PGMEA. To facilitate intact release of the small PDMS features of traps, the developed SU8 master was surface-treated with a fluorophilic coating by spin coating about 200 µl of 1:20 Cytop CTL-809M in CTsolv.100E (both from Asahi Glass Co. Ltd., Japan) onto the master. For this, the Cytop dilution was directly pipetted onto the SU8 features and then spin-coated at 3000 rpm for 1 min, using a 500 r.p.m./s ramp. The wafer was then hard-baked for 30 min at 453 K on a hot plate to simultaneously drive SU8 polymerization to completion whilst also covalently anchoring the coating. The master was then allowed to slowly cool down to room temperature by turning off the hot plate.

PDMS base and curing agent (Sylgart 184, Dow Corning) were mixed at a ratio of 10:1, and poured to about 4 mm thickness onto the master in a petri-dish. After degassing for about 15 min, PDMS prepolymer was cured on the silicone master at 80 °C for at least 1 h. The PDMS was then peeled off the silicon wafer, cut to size and fluid ports were punched with a 4 mm and 0.5 mm diameter biopsy puncher (World-Precision-Instuments) for the reservoir and outlet, respectively. The PDMS microchannels were then sealed by plasma bonding them onto glass cover slips (24 × 50 mm, VWR) using oxygen plasma (15 s at 0.3mbar, 30% power, Diener, ZEPTO) and bonded for 15–30 min at 80 °C. Before introduction of GUVs, the bottom layer channels were filled, via centrifugation (900 g, 10 min), with 1% (w/v) pluronic F-127 (Sigma) solution in PBS to coat the channels. This prevents vesicle rupture upon contact with the walls. After coating, a syringe-pump (neMESYS, cetoni, Germany) was used to draw the GUV solution and reagents through the fluid channels during the experiments using a flow rate of 4 µl/h.

### Confocal microscopy

Imaging was performed with an LSM 780/CC3 confocal microscope (Carl Zeiss, Germany) equipped with a C-Apochromat, 40 × /1.2 W objective. We used PMT detectors (integration mode) to detect fluorescence emission (excitation at 488 nm for YFP and 640 nm for DOPE-ATTO 655) and record confocal images, typically focusing on the equatorial plane of the GUVs. After some of the GUVs trapped on the chip for imaging, the buffer was pumped to the chip for about 10 min to remove small membranes in the background. Afterwards, individual GUVs were picked and focused. For the control experiments to quantify the stability of the GUVs, freshly sample buffer was loaded for 10 min and images of individual GUVs was taken every minute. For IFT172-Venus membrane-binding test, diluted IFT172 to 250 nM and 50 nM was loaded after buffer wash and images of individual GUVs was taken every minute. The osmolarity of all the buffers and diluted protein solutions were measured and matched (Fiske® Micro-Osmometer). All images were analyzed using ImageJ.

### Antibodies and cell culture

The following antibodies were used in this study: Anti-IFT172 (mouse monoclonal, sc-398393, Santa Cruz, used with 1:100 dilution), Anti-Arl13b (1711-1-A; Proteintech, 1:500 dilution). The secondary antibodies Alexa Fluor 488 mouse (A21202, 1:600 dilution) and Alexa Fluor 568 rabbit (A10042, 1:600 dilution) were purchased from Thermo Fisher Scientific. HeLa cells (ATCC) were grown in DMEM medium containing 10% FCS at 37 °C with 5% CO_2_. RPE-1 cells (ATCC) were grown in DMEM/F12 medium containing 10% FCS at 37 °C with 5% CO_2_.

### Cell fractionation assay

To identify IFT172 in subcellular compartments, RPE-1 cells (ATCC) were fractionated^35^. Cells from two 10 cm cell culture plates were collected in 4 ml lysis buffer (25 mM Tris-HCl, pH 7.5, 50 mM sucrose, 0.5 mM MgCl_2_, 0.2 mM EGTA), followed by homogenization with a Dounce homogenizer. The sucrose concentration was restored to 250 mM afterwards. Nuclear materials were removed by centrifugation at 1000 × g for 10 min (Multifuge 1 L, Heraeus). The supernatant was collected and further centrifuged at 195,000 xg for 30 min (Optima MAX Ultracentrifuge, Beckman Coulter) to separate cytosolic (supernatant) and organellar (pellet) fractions. The pellet was resuspended in SDS buffer (2.5% SDS, 50 mM Tris pH 8.1) for western blotting. Total cell lysate, cytosolic fraction and organellar fraction were separated by SDS-PAGE and analyzed with Western blot analysis. Anti-IFT172 antibody used with 1:100 dilution and goat anti-mouse IgG (62-6520, Thermo Fisher) was used as secondary antibody with 1:5000 dilution. Uncropped blots are available in Supplementary Figure 5.

### Immunofluorescence microscopy

RPE-1 and HeLa cells were cultured on glass cover slips were used for immunostaining. Cells were fixed with 4% formaldehyde (Thermo Fisher Scientific) for 10 min, washed with PBS for 3 times, followed by the permeabilization in 0.01% Triton X-100 for 15 min. After rinsing with PBS, the cover slips were blocked with 4% BSA for 30 min, rinsed again with PBS, and then incubate with primary antibodies diluted in PBS containing 4% BSA for overnight at 4 °C. The samples were rinsed three times with PBS containing 4% BSA and then incubated with secondary antibodies diluted in PBS containing 4% BSA for 1 h at room temperature. Slides were then washed and mounted with Prolong gold antifade reagent (Thermo Fisher Scientific) and checked at room temperature on CF2 Leica TCS SP8 microscope equipped with a 63 × 1.40 OIL lens. The images were merged and processed using Fiji^55^.

To overexpress Rab5-GFP or lysozyme C-mCherry in Hela cell for co-staining with IFT172, the plasmids encoding Rab5-GFP or lysozyme C-mCherry (plasmids obtained as gifts by Dr. Julia von Blume) was transfected into Hela cells followed with 24 h incubation at 37 °C.

### Dynamic light scattering

For measuring the size of the purified IFT172, dynamic light scattering was carried out on a DynaPro NanoStar Instrument (Wyatt) using 50-µl cuvettes and IFT172 volume of 20 µl at 4°C. Raw data were analyzed by the DYNAMICS software package. The dispersity of the solution was assessed, and the average hydrodynamic radius (RH) was calculated.

For measuring the size of the liposomes, a Malvern Zetasizer Nano ZSP system (Malvern, UK) was used using disposable micro cuvettes (ZEN0040; Malvern, Malvern, UK) with the backscatter (173°). For each experiment, 3 scans (6–10 runs each) were measured at 25 °C with an initial equilibration time of 3 min. Dispersant viscosity and refractive index (r.i.) were set to 0.8882 cP and 1.330, respectively, and the pre-defined settings for liposomes were used for the scattering material (abs 0.001, r.i. 1.330). The liposome concentration was 0.1 mg/ml in all experiments to allow measurements with an attenuator of 7 or 8 (mean adjusted count rate of ~20,000). For all measurements, the fits of the raw correlation data met the manufacturer’s quality criterion. The values plotted for each experimental condition are the mean size distributions for 3 runs as obtained from the instrument software (based on the measured intensities and the general purpose model (normal resolution)). The count rate for control measurements with IFT172 alone was ~10-fold lower than for experiments with liposomes, and the mean of the size distribution was at 64 ± 13 nm (S.D.), from 3 independent experiments. Therefore, we assess that the shift in the size distribution observed after protein addition is due to the absorption of the protein to the liposomes.

### Reporting Summary

Further information on research design is available in the Nature Research Reporting Summary linked to this article.

## Electronic supplementary material

Supplementary Information

Supplementary Movie 1

Source Data

Description of Additional Supplementary Files

Reporting Summary

## Data Availability

Data supporting the findings of this manuscript are available from the corresponding authors upon reasonable request. A Reporting Summary for this Article is available as a Supplementary Information file. The source data underlying Figs. 3c, e, 4e, h, and 7b are provided as a Source Data file.
