# Supplementary material for: Membrane association and remodeling by intraflagellar transport protein IFT172
Source: Nat Commun. 2018 Nov 8;9:4684. doi: 10.1038/s41467-018-07037-9 (PMC6224603; doi:10.1038/s41467-018-07037-9)
Supplement: Supplementary file 1 — Supplementary Information [file 41467_2018_7037_MOESM1_ESM.pdf]

Membrane association and remodeling by intraflagellar transport protein IFT172

Wang et al.

Supplementary information

Mass	MS Intensity (×10^4)	Matched Mass	Delta	Abbreviation	Formula
786.5990	4	786.6008	0.0018	PC, PE	$C_{44}H_{85}NO_8P$
758.5694	4	758.5695	0.0001	PC, PE	$C_{42}H_{81}NO_8P$
788.6149	3	788.6164	0.0015	PC, PE	$C_{44}H_{87}NO_8P$
760.5825	2	760.5851	0.0026	PC, PE	$C_{42}H_{83}NO_8P$
744.5524	2	744.5538	0.0014	PC, PE	$C_{41}H_{79}NO_8P$

Supplementary Table 1 - Mass spectrometry result of purified IFT172 shows co-purification with lipids.

The five most intense peaks are shown with molecular mass (Mass), and the intensity value (MS Intensity) detected by the mass spectrometry measurement. The mass was compared to known lipid masses using an online tool (http://www.lipidmaps.org/tools/index.html). “Delta” shows the difference between the input mass and the calculated mass. Abbreviation and Formula show the possible lipid species based on the molecular weight.

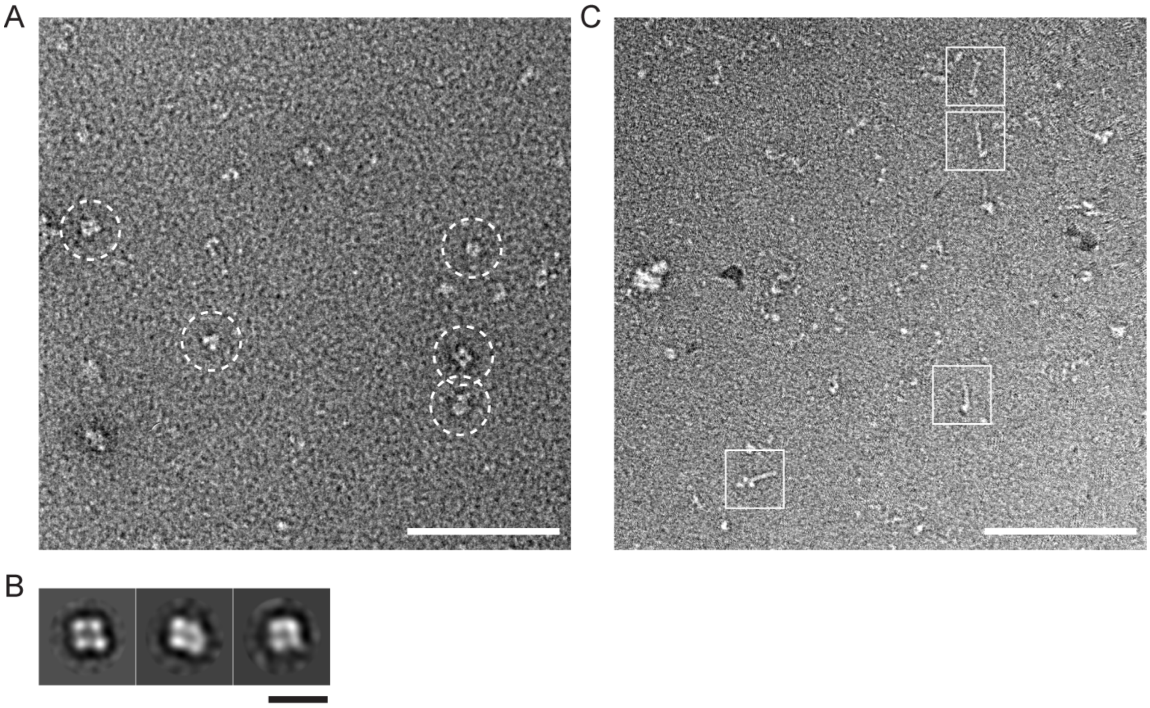

Supplementary Figure 1

IFT172 monomer purified with additional anion-exchange chromatography.

A and **B.** Negative-stain EM image (A) and 2D reference free class average of IFT172 monomer (B) shows the closed conformation without DDM during purification. Scale bars 100 nm in (A), and 20 nm in (B). **C** IFT172 employs the open conformation (marked with square) in the presence of DDM. Scale bar: 100 nm.

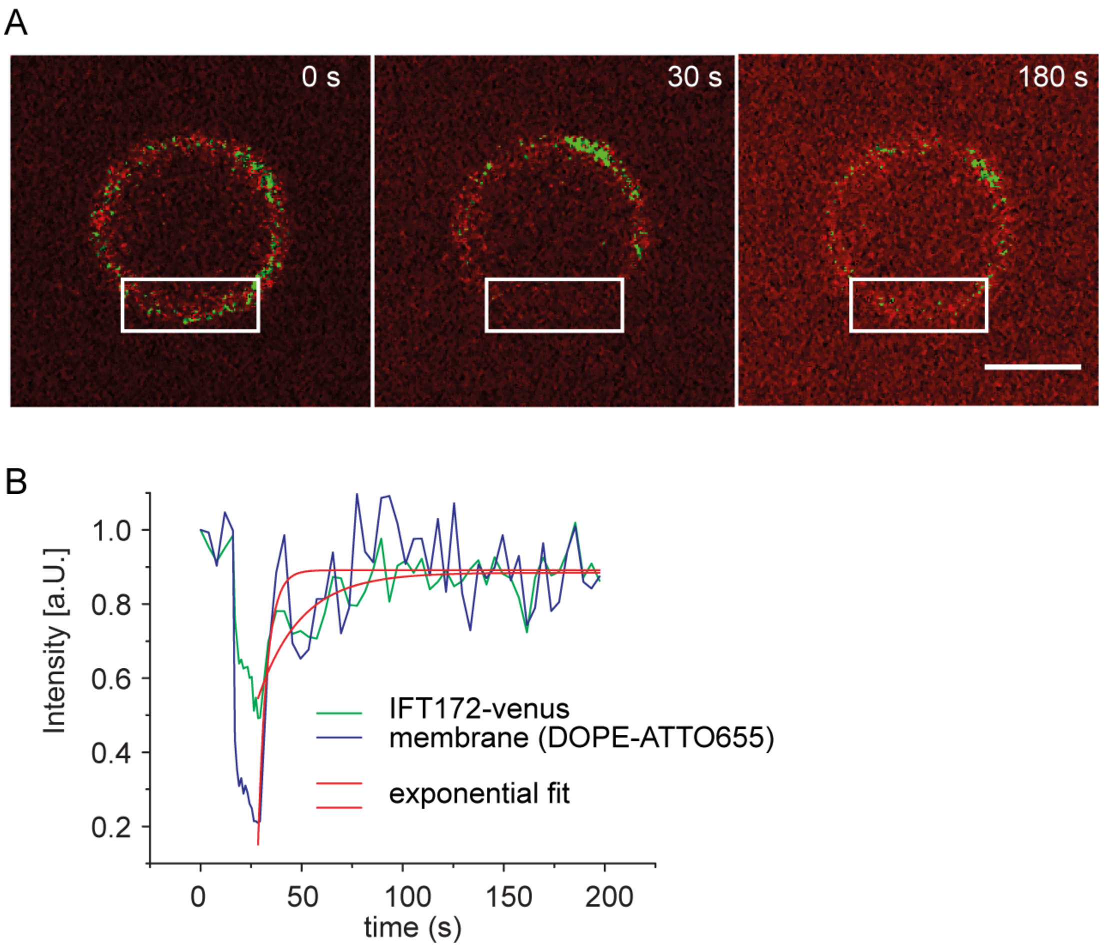

Supplementary Figure 2

A. Confocal image of IFT172-venus bound to the membrane of a GUV before (left) and after (middle) photobleaching the region marked in white. The right image shows the recovery of IFT172 fluorescence in the bleached region. Images were corrected for photobleaching and despeckled. Scale bar: 5 µm. **B.** Corresponding intensity traces of the FRAP experiment showing both mobility of membrane-bound IFT172-venus and the membrane itself (DOPE-ATTO655).

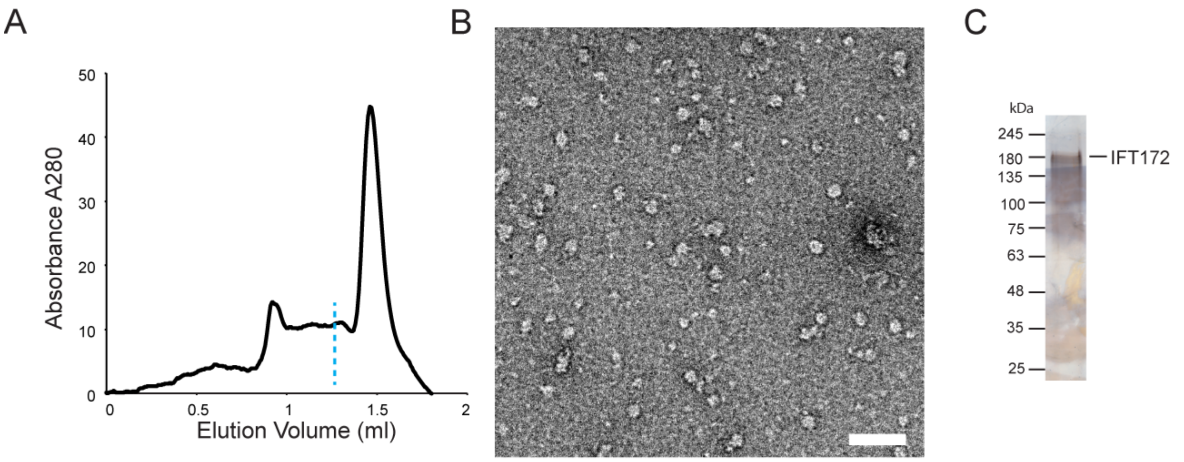

D

Mass	MS Intensity	Matched Mass	Delta	Abbreviation	Formula
1196.7183	3×10^3	1196.6105	0.1078	M(IP)2C	$C_{53}H_{100}NO_{24}P_2$
1197.7186	2.8×10^3	1197.7342	0.0156	PIP	$C_{64}H_{111}NO_{16}P_2$
1198.7156	2×10^3	1198.6262	0.0894	M(IP)2C	$C_{53}H_{102}NO_{24}P_2$

Supplementary Figure 3

Membrane remodeling products by IFT172 contain both IFT172 and lipids.

A. SEC profile of IFT172 with Folch Fraction I mixture. 10 µM of IFT172 was mixed with 2 mM Folch Fraction I and incubated for 10 min. Large lipid aggregates were removed by centrifugation before loading on the column. **B.** Negative staining micrograph of the fraction marked in A. Scale bar: 50 nm. **C.** Silver-stained gel of the fraction marked in A shows that IFT172 is present in the fraction. **D.** Mass spectrometric analysis of the fraction confirms the presence of lipids.

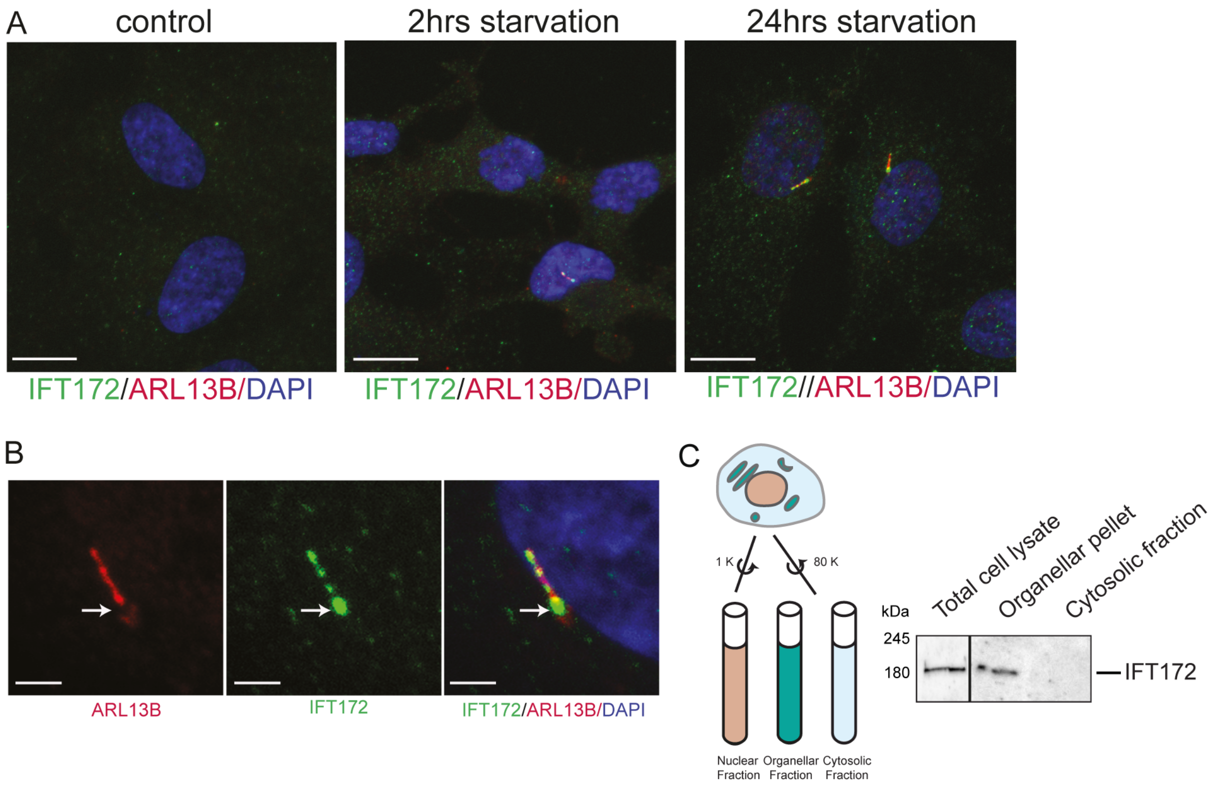

Supplementary Figure 4

Subcellular localization of endogenous IFT172.

A and **B.** Antibody staining of IFT172 in RPE-1 cells. IFT172 and Arl13b co-localization in the cilia in 24h-starved RPE-1 cells. Scale bar: A- 10 µm and B- 2 µm. ARL13b is labelled in red, IFT172 in green and DAPI in blue. **C.** Scheme of cell fractionation assay (left) and Western blotting of total cell lysate, organellar pellet, and cytosolic fraction of RPE-1 cell (right). Antibody was used at a dilution of 1:100.

number	primer name	sequence
MT262	CrIFT172 1-fw (+EcoRI)	GCGCGCGAATTCATGCAACTGCGTTACTTCAAATCAATCCTGC
MT262	CrIFT172 C-rev (+His; +XbaI)	GCGCGCTCTAGATTAATGGTGATGGTGATGGTGGTACATAGGTGCCGCCTGCATACCCG
MT319	CrIFT172 1-fw (+SmaI)	GCGCGCCCCGGGATGCAACTGCGTTACTTCAAATCAATCCTGC
MT320	CrIFT172 C-rev (+His, +SphI)	GCGCGCGCATGCTTAATGGTGATGGTGATGGTGGTACATAGGTGCCG
MT322	CrIFT172 590-fw (+SmaI)	GCGCGCCCCGGGATGAATACGGTGTCCTACGCACTGGATGAAG
MT391	CrIFT172 968-rev (+His, +XbaI)	GCGCGCTCTAGATTAATGGTGATGGTGATGGTGGTAACCGCGCGCGACACGCTG
MT878	CrIFT172 C-rev (noStop, +XbaI)	GCGCGCTCTAGAGTACATAGGTGCCGCCTGCATACCC
MM013	CrIFT57 1-fw	CCAGGGAGCAGCCTCGATGATGAGCAGCAAGCGGGGTGGGCG
MM014	CrIFT57 C-rev	GCAAAGCACCGGCCTCGTTAGTCCTCCTCCTCGTCACTGAGAGCC

Supplementary Table 2 – Primers used for this study.

Supplementary figure 4C

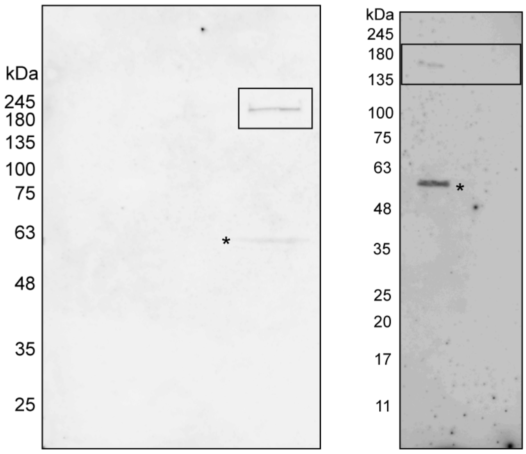

Supplementary Figure 5

Original uncropped Western blots.

Squared bands are used for display in Supplementary Figure 5. " * " indicates partially degraded IFT172.
