# Supplementary material for: Membrane association and remodeling by intraflagellar transport protein IFT172
Source: Nat Commun. 2018 Nov 8;9:4684. doi: 10.1038/s41467-018-07037-9 (PMC6224603; doi:10.1038/s41467-018-07037-9)
Supplement: Supplementary file 4 — Description of Additional Supplementary Files [file 41467_2018_7037_MOESM4_ESM.pdf]

Title: Supplementary Movie 1

Description: Confocal microscopy showing the coating of IFT172 (left) on a GUV surface (right). The movie was recorded with 20 s each frame. “
